# Metal Oxide-Functionalized Photopolymers: A Perspective in 3D Printing

**DOI:** 10.1021/acspolymersau.5c00065

**Published:** 2025-09-22

**Authors:** Martina Korčušková, Petr Lepcio, Josef Jančář

**Affiliations:** 1 Central European Institute of Technology, 48274 Brno University of Technology, Purkyňova 656/123, 612 00 Brno, Czech Republic; 2 Institute of Materials Science, Faculty of Chemistry, 48274 Brno University of Technology, Purkyňova 118, 612 00 Brno, Czech Republic

**Keywords:** polymer nanocomposites, photocatalytic activity, nanoreinforcement, dispersion stability, light absorption and scattering, thermal stability, dielectric properties, antimicrobial activity, biocompatibility

## Abstract

Vat photopolymerization is a widely adopted additive manufacturing technique valued for its high resolution, smooth surface finish, and rapid production speed. Recently, it has gained prominence in the fabrication of polymer nanocomposites, as liquid photopolymer resins allow efficient incorporation and dispersion of nanoparticles. Current research in vat 3D printing of polymer nanocomposites is directed toward creating materials with enhanced functionalities, enabling the development of advanced functional components. Among different nanofillers, semiconducting metal oxide nanoparticles (MOx NPs) such as TiO_2_, ZnO, Fe_3_O_4_, Cu_2_O, and ZrO_2_ are of particular interest. These NPs act not only as functional additives but also as photocatalysts, directly influencing photopolymerization kinetics, cross-linking density, and final properties. Mechanical performance is enhanced through nanoreinforcement, provided that homogeneous NP dispersion is achieved. This enables lightweight, high-performance parts for aerospace, automotive, and biomedical engineering. MOx NPs also improve thermal stability, supporting applications in electronics, automotive systems, and energy devices. Adjustments in electrical and dielectric properties open further potential in power electronics, high-voltage insulation, and wearable devices. Incorporation of superparamagnetic Fe_3_O_4_ introduces magnetic functionality, useful for microactuators, sensors, and graded materials. Optical properties can likewise be tailored, with MOx/polymer nanocomposites enabling photodetectors, optoelectronic components, and functional thin films. In the biomedical field, biofunctional performance—ranging from antimicrobial activity to tissue compatibility—has been exploited in dentistry, tissue scaffolds, and micromachines for drug delivery. Despite these advances, challenges such as nanoparticle aggregation, viscosity increase, light scattering, and altered reaction kinetics still limit the achievable filler loadings and overall performance of vat-printed nanocomposites. This review therefore emphasizes both the potential and the limitations of incorporating MOx nanoparticles into vat photopolymerization, outlining the current state of knowledge and key challenges that must be addressed to enable application-oriented functional materials.

## Graphical abstract

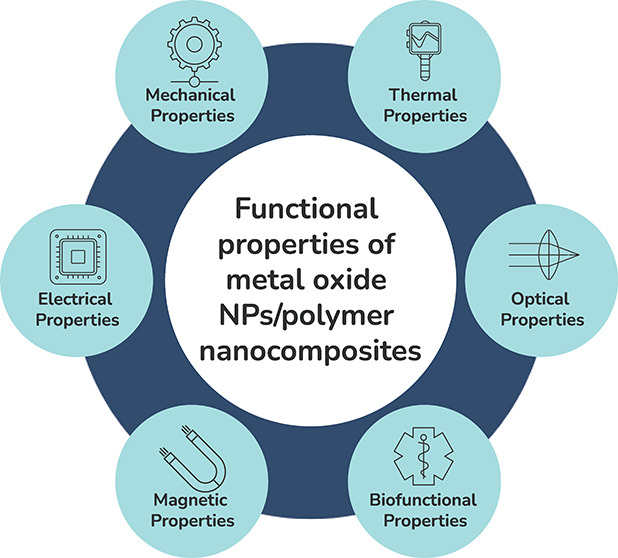

## 1 Introduction

Vat 3D printing, also known as vat photopolymerization, is a widely utilized additive manufacturing technology recognized for its high resolution and printing efficiency.^1–11^ In this method, a liquid photopolymer resin is selectively cured through targeted light-activated polymerization. The resin is contained in a vat, with the build platform submerged in the liquid. When exposed to specific light wavelengths, the liquid photopolymer undergoes rapid photopolymerization, transforming into a solid state.^11–16^

Despite its advantages, such as high precision, fast printing, and minimized material waste, vat 3D printing has some limitations. These include high acquisition costs, limited material choices, UV light sensitivity, and layered manufacturing patterns.^17–20^ There are various vat 3D printing technologies, with stereolithography (SLA), masked SLA (M-SLA), and digital light processing (DLP) being the most prominent. All share the core principle of selectively curing a liquid photopolymer resin layer-by-layer to produce 3D objects with high accuracy and smooth surface finishes (Figure 1).^11–13,21–23^

**Figure 1:**
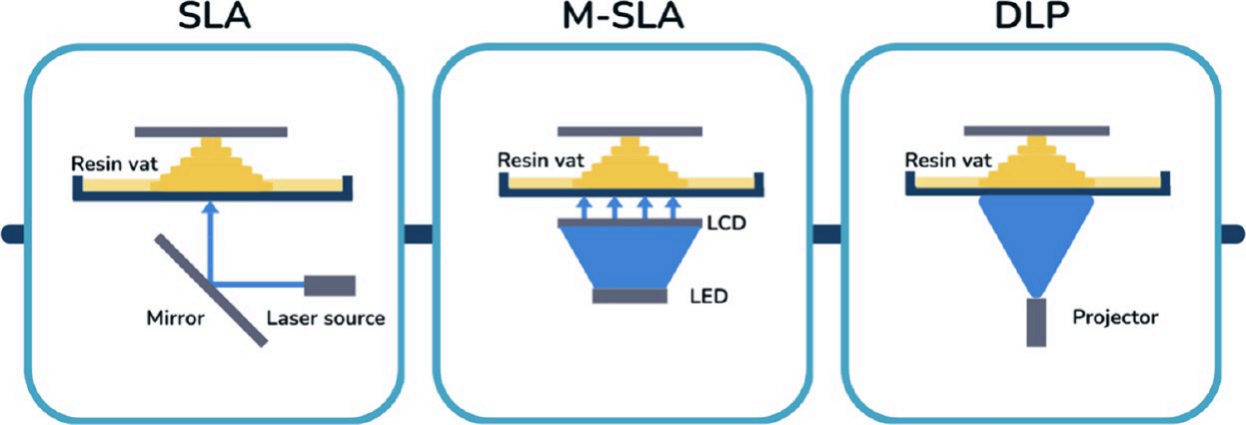
Schematic comparison of vat photopolymerization techniques: SLA uses a laser and mirror to cure resin point-by-point; M-SLA employs an LCD mask with a light source to cure entire layers; DLP projects light through a digital micromirror device (DMD) to cure layers simultaneously. Adapted from ref 24. Available under a CC-BY 4.0 license. Copyright 2024 Chekkaramkodi et al.

Recently, there has been a growing demand for the development of polymer nanocomposites with enhanced functional properties. These include mechanical,^25–29^ thermal,^27,30–32^ electrical,^33–38^ magnetic,^39–42^ optical,^34,43–46^ and biofunctional^39,47–52^ characteristics, enabling their application across a wide range of high-tech industries, as illustrated in Figure 2 for metal oxide NPs/polymer nanocomposites. To meet these requirements, diverse filler materials are incorporated into polymers to enhance their functional properties.^53,54^

**Figure 2:**
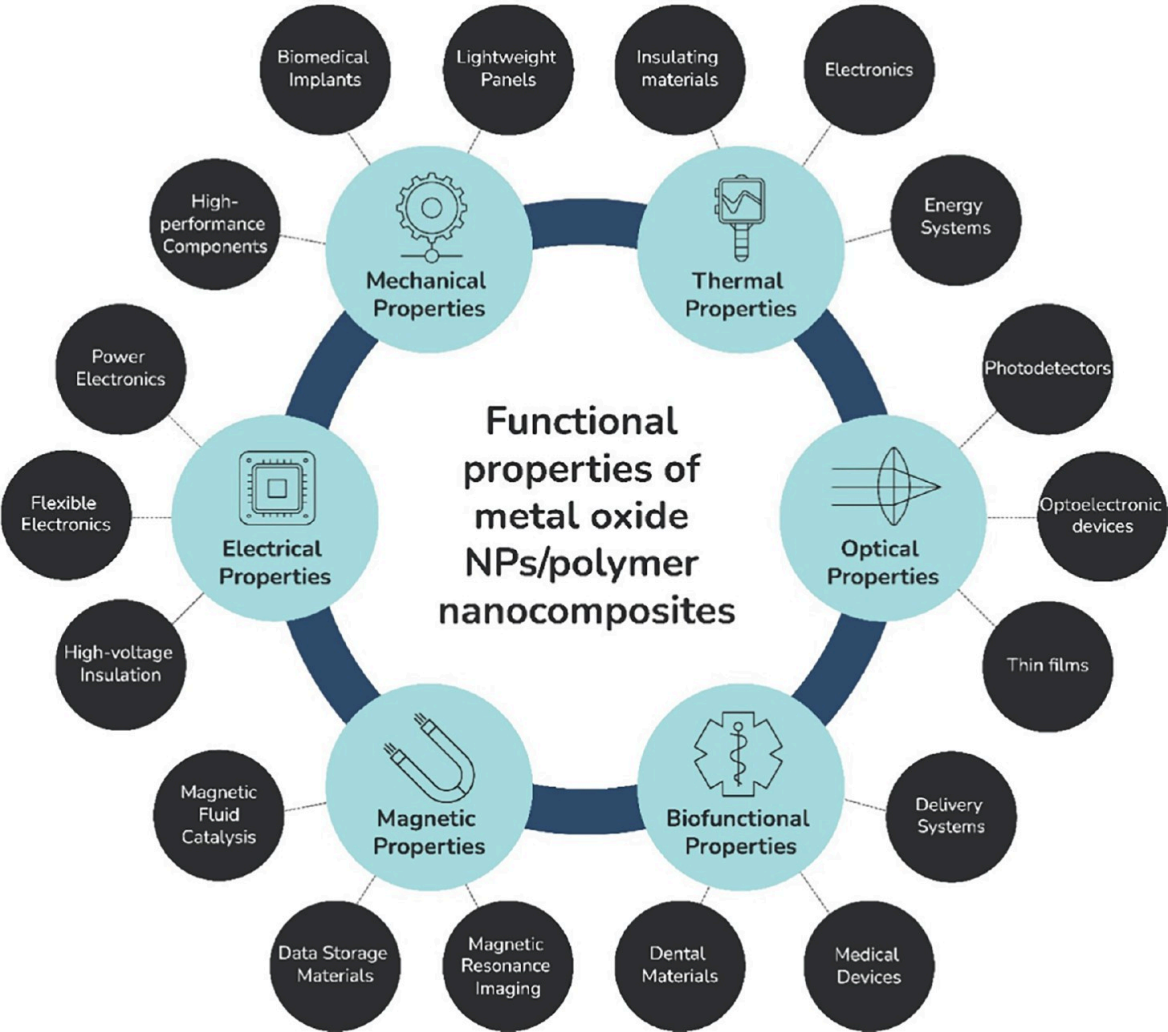
Overview of functional properties of metal oxide nanoparticles/polymer nanocomposites and their application areas.

Given the nature of the photopolymerization process, the fillers used are typically nanosized to ensure efficient dispersion and polymer network integration. Commonly employed nanofillers include carbon-based materials (carbon nanotubes, graphene, fullerenes), metallic nanoparticles (gold, silver, or copper NPs), metal oxide nanoparticles (titanium dioxide, zinc oxide, iron oxide NPs), and ceramic nanoparticles (clay, silica NPs). Polymeric nanoparticles (dendrimers, polymeric micelles), quantum dots, and hybrid nanoparticles are also utilized.^53–61^ The incorporation of nanofillers enhances various properties, including mechanical strength, thermal and UV stability, biocompatibility, and customized functionality for specific applications.^62–68^

Incorporating nanoparticles (NPs) into polymer resin for vat 3D printing presents several challenges, which are summarized in Table 1. Addressing all these challenges requires careful selection of material composition, nanocomposite preparation methods, and 3D printing parameters. Achieving the desired functional properties requires optimizing various factors to strike a balance between good printability and the highest possible NP concentration, as these NPs are the primary contributors to the material’s functional characteristics.

**Table 1:** Major Drawback Associated with Incorporating Nanofillers into the Vat 3D Printing Process

**Drawback**	**Description**	**ref.**
Aggregation and agglomeration	NPs tend to agglomerate due to their high surface area-to-volume ratio and van der Waals forces, impacting the material’s final properties. Achieving homogeneous dispersion maximizes the surface area of NPs and minimizes the distance between them, enhancing interactions with polymer chains. Proper dispersion is achieved through intensive mechanical mixing, shear mixing, sonication, ultrasonication, or their combinations.	^37^,^56^,^57^,^62^,^64^,^69–73^
	NP agglomeration can be further reduced by surface modification, which also increases interfacial bonding. Silane coupling agents are commonly used because they possess functional groups that bridge hydrophilic NPs and hydrophobic polymers. NPs may also be stabilized by polymeric surfactants that adsorb onto the particle surface and provide electrostatic or steric barriers against reagglomeration. Surface modification improves not only interfacial bonding but also mechanical properties, electrical conductivity and thermal stability, because the attachment of specific functional groups to NP surfaces creates an interphase and simultaneously introduces additional chemical functionality into the system. In combination with appropriate physical dispersion, these chemical treatments produce a stable, well-dispersed distribution of NPs within the polymer matrix.	
Viscosity control	The incorporation of NPs increases resin viscosity, which is critical for printability. Moreover, it was found that dispersed NPs increase the viscosity considerably more than nondispersed systems. The viscosity of the resin should ideally range between 0.1–10 Pa·s for optimal printability. Excessively high viscosity can slow the printing process and increase the likelihood of defects due to uneven resin flow. Additionally, viscosity influences NP sedimentation within the polymer resin. While individual NPs undergo Brownian motion that generally counteracts sedimentation, agglomeration and aggregation can lead to the formation of larger clusters prone to settling. Consequently, low resin viscosity accelerates sedimentation unless the NPs are well dispersed and effectively stabilized.	^74–81^
Interfacial interactions	The distribution of NPs within the polymer matrix depends on the strength and nature of the interfacial interactions due to the polymer chain adsorption on the NP surfaces. These interactions may be noncovalent (such as the physical adsorption of polymer chains onto the NP surface) or covalent (the formation of chemical bonds between functional groups on the NPs and the polymers). Favorable polymer–NP interactions promote uniform dispersion and lower the percolation threshold, thereby enhancing the functional properties.	^64^,^76^,^82–85^
	Interfacial interactions can be enhanced by surface modification of NPs, which alters polymer–NP affinity through reactions with surface groups or by introducing layers with different dielectric properties or hydrophobicity. This approach is mainly used to compatibilize inherently hydrophilic NPs with polymers and improve dispersion; however, in nanocomposites with naturally strong polymer–NP interactions (e.g., hydrogen bonding), surface modification may negatively affect dispersion.	
Light absorption and scattering	Many NPs possess high light absorbance, which can hinder the effective interaction between light and photoinitiators, thereby slowing down or preventing the curing reaction. Additionally, nanoparticles exhibit considerable light scattering, especially when aggregated, leading to a decrease in the resolution of 3D printing. This interaction may introduce additional physical and chemical effects on the overall reaction. As a result, only a very low concentration of NPs can be usually utilized to maintain the efficacy of the photocuring reaction.	^1^,^66^,^74^,^86–88^
Altered reaction kinetics	NPs in the resin can alter the photopolymerization reaction kinetics. Many NPs, particularly metal oxides, can interact with the photoinitiators or absorb light themselves, thus altering the rate of polymerization. This change in kinetics can lead to an uneven curing process, potentially affecting the structural integrity and mechanical properties of the final printed part.	^66^,^86^,^89–91^
Defects in printed parts	Defects in vat-printed nanocomposites arise from the combined effects of nanoparticle aggregation, viscosity changes, interfacial interactions, and light absorption/scattering. Poor dispersion or NP clustering generates local heterogeneities that act as stress concentrators and promote crack initiation under load. Increased viscosity slows resin flow and layer recoating, leading to voids, incomplete layer filling, and weak bonding between successive layers. Locally accelerated curing around NP surfaces can form microgel regions with a higher cross-linking density than the bulk, resulting in residual stresses and brittleness. Light absorption and scattering reduce cure depth and printing resolution, yielding poorly defined features and rough surface morphology. Collectively, these effects compromise dimensional accuracy, mechanical integrity, and overall quality of the printed components.	^1^,^62^,^69^,^70^,^92–94^

Postprocessing significantly influences the properties of vat 3D-printed parts. Freshly printed parts often contain uncured resin, requiring cleaning (e.g., with isopropyl alcohol) and additional curing. Postcuring promotes further cross-linking of the polymer network, enhancing mechanical properties. Curing is carried out either by placing the part under UV light or by heating it in an oven, depending on the material used.^74,95^ The effect of postprocessing on final properties was studied by Štaffová et al.,^65^ who reported that curing time and total exposure energy significantly influence the mechanical properties of 3D printed parts. Extended curing times and higher exposure doses result in an increased glass transition temperature and a shift of the entire glass transition region to higher temperatures. Additionally, these effects systematically scale with the network density which increases with reaction conversion. The absorption and scattering of curing light by NPs in polymer nanocomposites are expected to impact the efficiency of the postcuring process, although this effect has not been thoroughly described yet.

Vat 3D printing is a promising technique for the convenient fabrication of functional nanocomposite systems. While extensive research has explored the preparation and performance of nanocomposites, most studies have predominantly focused on carbon-based nanofillers. To the best of our knowledge, no comprehensive review has specifically examined the influence of semiconducting metal oxide nanoparticles on the functional performance of polymer nanocomposites. This study addresses this gap by providing a detailed analysis of the functional properties of semiconducting metal oxide NPs/polymer nanocomposites prepared through the vat 3D printing process.

## 2 Photocatalytic Metal Oxide Nanoparticles and Their Impact on the Vat 3D Printing Performance

Semiconducting metal oxide nanoparticles (MOx NPs) have become essential additives in vat 3D printing, significantly enhancing the performance and functionality of polymer nanocomposites. This group includes NPs such as titanium dioxide (TiO_2_), zinc oxide (ZnO), iron oxide (Fe_3_O_4_), copper oxide (Cu_2_O), aluminum oxide (Al_2_O_3_) and silver oxide (Ag_2_O), among others.^96^ Certain MOx NPs exhibit photoactivity, enabling them to function as photoinitiators. This property is particularly advantageous, as many commercially used free-radical photoinitiators are associated with significant cytotoxicity.^97^ By incorporating these NPs into the vat 3D printing process, they can facilitate photoinitiation, potentially minimizing the reliance on traditional photoinitiators (PIs) and contributing to a more sustainable approach. Furthermore, unlike conventional PIs that are depleted during photopolymerization and generate harmful byproducts, MOx NPs generate radicals through a photocatalytic cycle. This process can lead to additional polymer branching and cross-linking, which may enhance the mechanical properties of the system compared to traditional PI-mediated photopolymerization.^98–100^

Photoactivity refers to the ability of certain semiconductors to undergo chemical reactions when exposed to light of sufficient energy. Semiconductors typically have a wide band gap, with separated valence band (VB) and conduction band (CB) (Figure 3). When photons with energy equal to or greater than the semiconductor NP’s band gap interact with atoms, electrons (e^–^) in the VB are excited to the CB, leaving behind a hole (h^+^) in the VB. This process generates electron–hole pairs that can diffuse through the material, leading to trapping or recombination. The generation, diffusion, trapping, and recombination of these charge carriers in response to irradiation are known as primary photoelectric processes. Furthermore, these generated charge carriers can be transferred from the photoactive particles to electron acceptors or donors, such as acrylic monomers, enabling the photoactive particles to function as photoinitiators.^101–104^

**Figure 3:**
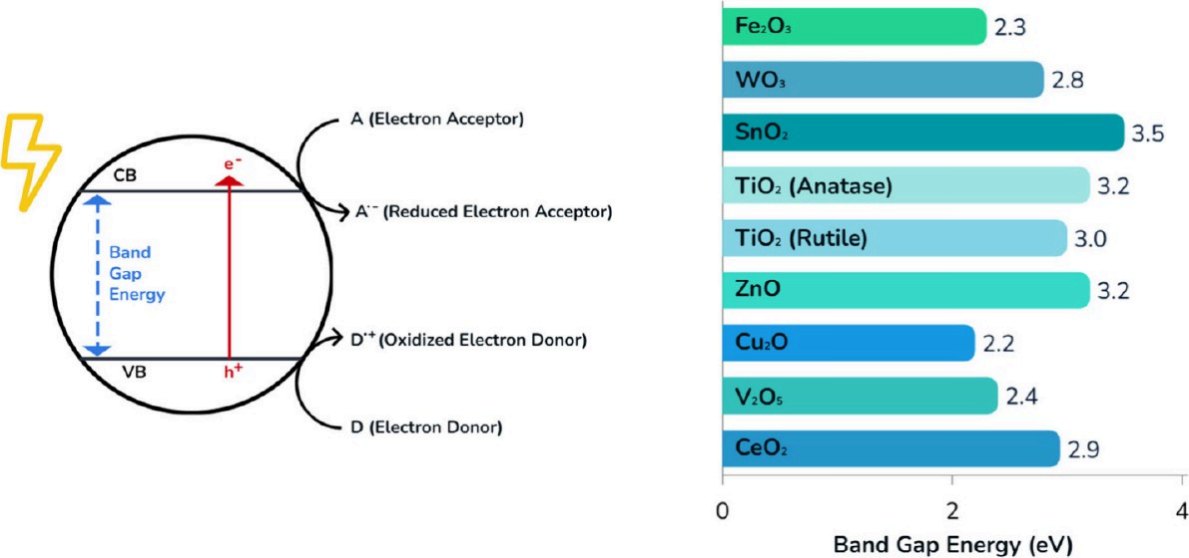
Illustration of the photocatalytic mechanism of metal oxide semiconductors showing band gap excitation and charge transfer, along with the band gap energies of selected metal oxides. Adapted from ref 86. Available under a CC-BY 4.0 license. Copyright 2024 Korčušková et al. and adapted with permission from ref 105. Copyright 2016 Elsevier.

The most extensively studied photoactive metal oxide is TiO_2_, which is also widely used as a photocatalyst in environmental applications. The photogenerated free electrons and holes can react with adjacent molecules near the surface, such as O_2_, H_2_O and OH^–^, generating reactive oxygen species (ROS), namely superoxide anion (•O_2_^–^) and the hydroxyl radical (•OH) which subsequently form hydrogen peroxide (H_2_O_2_).^106^ These surface reactions play a crucial role in the photodegradation of environmental contaminants.^101,107–109^ Analogical mechanism can be applied in vat 3D printing, where the generated charge carriers interact with the monomers and initiate polymerization.

The key factor determining photocatalytic capabilities is the band gap energy. The band gap energies of various metal oxides correspond to visible or UV light (Figure 3). Commercial vat 3D printers typically utilize LED lamps with a peak emission wavelength of 405 nm,^110^ although the emitted light spans a broader spectrum.^65^ Consequently, the photocatalytic activity of metal oxide semiconductors becomes particularly significant when their band gap energies correspond to 3.1 eV, or lower.^111^ Nevertheless, the exact value is influenced by factors such as the NP size and shape, oxide composition (e.g., impurities), surface molecule adsorption, and doping with other metals. The band gap size can also vary in solutions due to the formation of the electrostatic double layer and ligand adsorption.^112,113^

Regarding size, MOx NPs with diameters smaller than 20 nm exhibit a larger band gap compared to larger NPs or bulk material. These differences are explained by quantum mechanics. While Fermi level, the highest occupied electronic energy level at 0 K, is continuous in bulk materials, it becomes discreet in subnanosized materials. A higher Fermi level allows more electrons to transition from the valence band to the conduction band, playing a crucial role in material conductivity. In conductors, the Fermi level lies within the conduction band, while in semiconductors, it is situated within the band gap.^112,114^ Other characteristics, such as crystal structure and the presence or type of defects, also change with particle size and can influence the formation rate of ROS, where reduced number of defects in NPs has been linked to lower ROS formation.^115^

Several studies have focused on monitoring the influence of TiO_2_ NPs on the kinetics and course of the photopolymerization reaction. Li et al.^116^ investigated the impact of rutile-form TiO_2_ NPs on the kinetics of epoxy acrylate resin. Adding 4 wt. % of TiO_2_ NPs accelerated the initial polymerization rate, but attributed to the gel effect and, consequently, slowed down the photopolymerization due to vitrification. Additionally, the overall double bond conversion decreased with the addition of NPs to the resin, which was attributed to the UV absorption by TiO_2_ NPs in the same wavelength range as PI, thereby reducing the PI’s efficiency. Becker-Willinger et al.^117^ used photo-DSC measurements to monitor the photoactivity of TiO_2_ in its anatase form. They found that adding up to 2.5 wt. % of TiO_2_ NPs to the acrylate-based resin accelerated the curing reaction kinetics. However, at a 5 wt. % concentration of TiO_2_ NPs, no reaction was observed, likely due to the dominant UV-absorbing properties of TiO_2_ NPs.

Luu et al.^118^ investigated the impact of TiO_2_ NPs on the kinetics of nanocomposite formation in the synthesis of hybrid materials. These materials consist of inorganic nanoblocks embedded within a polymer matrix, presenting potential applications in micro-optical devices. The matrix employed was poly­(2-hydroxyethyl methacrylate) (pHEMA). Raman spectroscopy revealed that increasing NP content reduces initiation and propagation rates and decreases the maximum conversion of C = C double bonds. This effect was attributed to both the UV absorption of TiO_2_ NPs and the formation of microgel regions around the NPs, hindering polymer chain bonding with surface ligands.

In addition to the studies mentioned above, our recent work^86^ provides deeper insights into the role of TiO_2_ NPs in both anatase and rutile forms on photopolymerization kinetics under different photoinitiating mechanisms and curing light wavelengths. The results demonstrate that both anatase and rutile exhibit photocatalytic activity, where free charge carriers directly initiate free-radical polymerization. However, their effectiveness is influenced by factors such as band gap energy, curing wavelength, light absorption efficiency, and electron transfer efficiency. At 405 nm, rutile was found to enhance monomer conversion and reaction kinetics at concentrations up to 1 wt. % (Figure 4A), while anatase outperformed rutile at 365 nm due to its lower recombination rate of charge carriers. In contrast, the interaction of TiO_2_ NPs with cationic photopolymerization followed a different mechanism. In this case, the TiO_2_ NPs did not directly photoinitiate the reaction but acted as photosensitizers. Specifically, they absorbed light to form excited species, which subsequently created an excited complex with the cationic photoinitiator, facilitating the initiation of the reaction. Anatase exhibited a more pronounced photosensitizing effect compared to rutile, while rutile had only a limited impact on final conversion and curing rate (Figure 4B).

**Figure 4:**
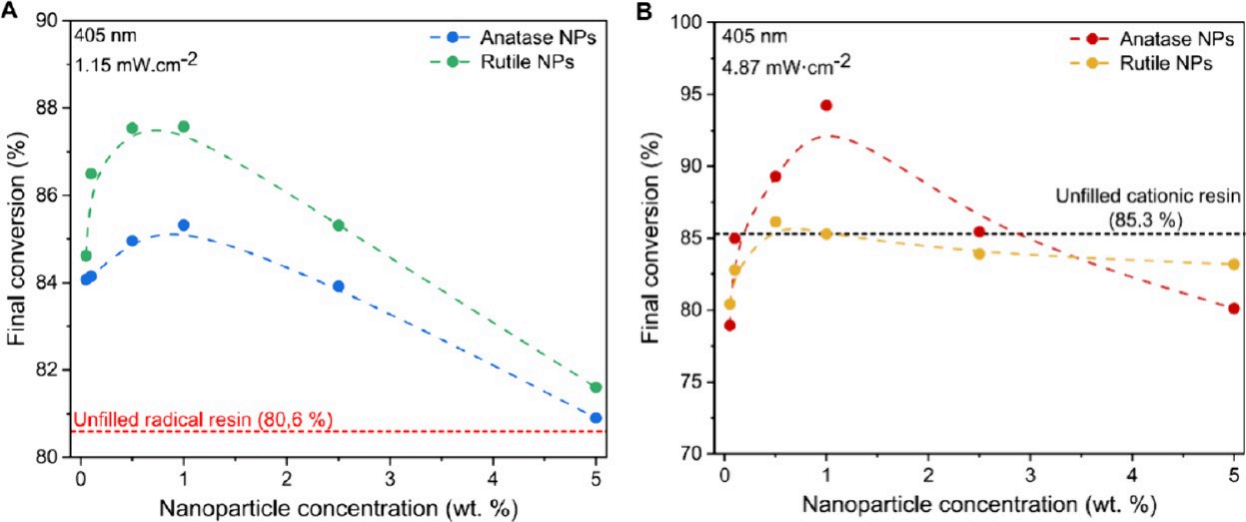
Effect of TiO_2_ nanoparticles on photopolymerization kinetics. (A) Final conversion of carbon-carbon double bonds in radical resin and (B) cationic resin as a function of nanoparticle concentration at 405 nm, with respective light intensities of 1.15 mW·cm^–2^ and 4.87 mW·cm^–2^. Dashed lines serve as visual guides. Reprinted from ref 86. Available under a CC-BY 4.0 license. Copyright 2024 Korčušková et al.

Apparently, TiO_2_ NPs exhibit a dual effect: they accelerate initial polymerization and cure reaction at lower concentrations but hinder overall reaction efficiency and double bond conversion at higher concentrations due to their UV-absorbing nature and propensity to form highly cross-linked regions. Therefore, optimizing both the concentration and dispersion of TiO_2_ NPs is essential to enhance photopolymerization kinetics and attain the desired properties in nanocomposite materials.

Zinc oxide (ZnO) is the second most researched photoactive MOx, after TiO_2_. Riad et al.^119^ explored the photocatalytic properties of TiO_2_ and ZnO NPs in an epoxide-based photopolymer, monitoring reaction kinetics using Fourier transform infrared spectroscopy (FTIR) and chemical titration. Their results indicated a faster reaction rate with TiO_2_ NPs, highlighting their superior photocatalytic efficiency compared to ZnO. Nonetheless, both types of NPs proved effective as photoinitiators in epoxy photopolymerization.

Naor et al.^100^ employed ZnO nanorods as quantum photoinitiators in an acrylate-based polymer system. These nanofillers acted as catalytic radical initiators, leading to nearly full polymer conversion (90% after 6 min) as observed by FTIR, without the need for additional PIs. Furthermore, ZnO nanorods were successfully utilized in a DLP 3D printer equipped with a 385 nm light source, fabricating woodpile structures with precise and well-defined features, thereby demonstrating the efficient photoinitiation capability of ZnO nanorods.

Further studies have investigated the impact of ZnO and alumina-doped zinc oxide (AZO) semiconducting NPs on the performance of 3D printing photopolymer resins.^66^ A key distinction between these two NPs was the characteristic surface plasmon resonance band of ZnO NPs at 377.5 nm, which was suppressed by alumina doping in AZO NPs. Consequently, AZO NPs enhanced conversion rates due to their photoactivity, with the fastest photopolymerization kinetics observed at loadings between 0.01 and 0.1 vol. %. Conversely, ZnO NPs reduced the conversion rate but increased the detected cure heat due to their photothermal effect.

Analogical effects were reported in functional MOx, such as magnetic Fe_3_O_4_ and CoFe_2_O_4_ NPs. The incorporation of these NPs significantly reduced the conversion degree of acrylate double bonds in polyurethane acrylate resin, as these NPs strongly absorb in the UV range, competing with the photoinitiator for UV light absorption.^120^ At the highest tested concentrations, 6.3 wt.% Fe_3_O_4_ resulted in a conversion degree of 20%, while 8.2 wt.% CoFe_2_O_4_ led to a conversion degree of 26%. However, when comparing these nanoparticles at similar concentrations, it is evident that modifying Fe_3_O_4_ with cobalt to form CoFe_2_O_4_ enhances the UV curing process, leading to a faster and more complete polymerization with a higher conversion degree. Specifically, Fe_3_O_4_ (6.3 wt.%) achieves a conversion degree of 20%, whereas CoFe_2_O_4_ (5.7 wt.%) reaches 45%. Additionally, the photopolymerization rate is improved, along with an increase in the maximum allowable filler content.^120^

While extensive research has highlighted the dual role of TiO_2_ and ZnO NPs in photopolymerization, acting both as photocatalysts and UV absorbers, the influence of other metal oxide NPs remains less well understood. Although some studies have explored photopolymerization in the presence of various MOx NPs, such as Cu_2_O^121^ or Fe_3_O_4_-Ag hybrid NPs,^122^ their impact on the kinetics and progression of the photopolymerization reaction remains insufficiently characterized.

## 3 Functional Properties

### 3.1 Mechanical Properties of Polymer Nanocomposites

Understanding the mechanical behavior of MOx NP–reinforced polymer nanocomposites is essential for their use as lightweight, high-performance components capable of meeting the stringent requirements of modern technologies, including high strength, thermal stability, low thermal expansion, and superior wear and corrosion resistance.^123–127^ Such materials are particularly attractive in aerospace and automotive engineering for lightweight yet durable structures that improve fuel efficiency and performance (e.g., wings, fuselages, cockpit interiors, seat covers),^37,126^ as well as in biomedical applications for the fabrication of orthopedic implants.^128–130^

Incorporating NPs with a large specific surface area into polymer matrices results in various distinct molecular processes due to interactions between polymer chains and NP surfaces. A key outcome of these interactions is nanoscale reinforcement, which significantly enhances the mechanical properties of the resulting nanocomposites. Jančář et al.^131^ attributed this effect to polymer–particle interfacial interactions. An interphase layer around each NP consists of two parts: immobilized and frustrated polymers. The immobilized layer comprises tightly adsorbed polymer segments at the NP surface, while the frustrated layer consists of polymers connected or intertangled with the immobilized layer. In both parts, the dynamics of polymer chains are slowed down, with a greater degree of retardation occurring in the immobilized layer. Ghanekarade et al.,^132^ linked this effect to short-range caging by the nearest neighbors and longer-range cooperative elastic displacement outside the nearest cage. The incorporation of NPs thus leads to the restricted mobility of polymer chains, resulting in improvements in material properties such as shear and bulk modulus, yield stress or toughness, especially above the *T*_g_.

The mobility changes in the area surrounding NPs were investigated by Ondreáš et al.,^62^ who measured variations in *T*_g_ values as a function of the volume fraction and spatial organization of NPs. Generally, polymer chains with retarded dynamics exhibit increased *T*_g_ values, while those with accelerated dynamics show lower *T*_g_. In this study, the most significant increase in *T*_g_ was observed in polymer nanocomposites with individually dispersed NPs. Initially, *T*_g_ values showed a rapid increase compared to the neat polymer, followed by slower growth at higher NP concentrations. Systems with clustered NPs exhibited a gradual rise in *T*_g_ with increasing NP volume fraction, while the lowest difference in *T*_g_ values was found in nanocomposites with aggregated NPs. However, all types of dispersion fell onto a single master curve when related to the average proximity of the nearest particle instead of the simple concentration dependence (Figure 5A). Therefore, good dispersion is crucial for effective utilization of NPs, yet it may be unachievable at higher NP concentrations due to aggregation tendencies.^62,83^

**Figure 5:**
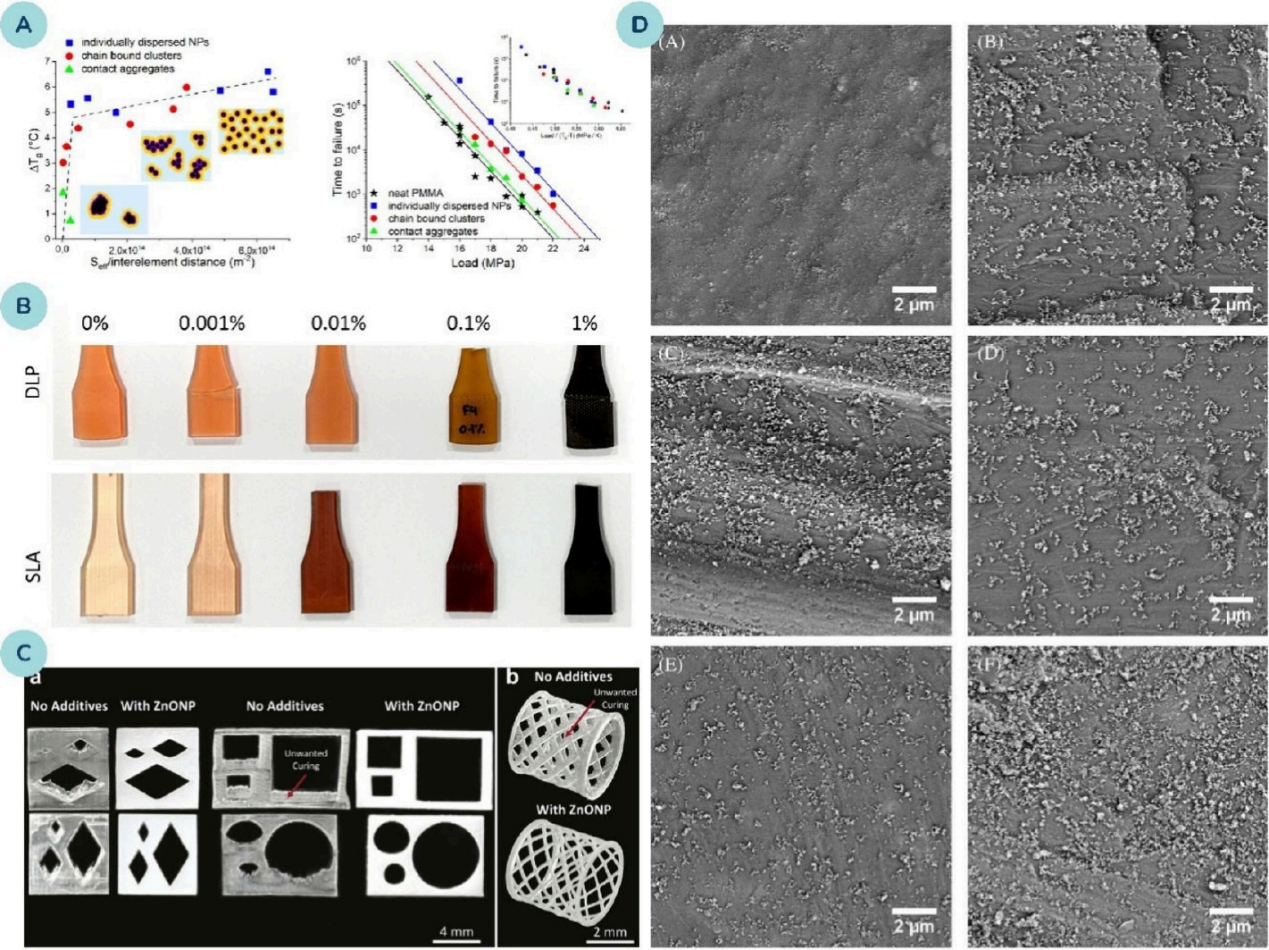
**(A)** The left part illustrates the change in glass-transition temperature (T_g_) as a function of the mean proximity of polymer chains to the nearest nanoparticle surface, forming a master curve for different dispersion types (individual particles, aggregates, clusters), while the right part shows the time to failure of those nanocomposites under applied load and forms a master curve when plotted against the change in T_g_ (inset). Reprinted with permission from ref 62. Copyright 2016 American Chemical Society. **(B)** Examples of printed material with varying concentrations of silver oxide. Reprinted from ref 133. Available under a CC-BY 4.0 license. Copyright 2024 Shannon et al. **(C)** DLP 3D-printed structures of photocurable resin, with and without ZnO nanoparticles, demonstrating the positive influence of nanoparticles on curing behavior and dimensional accuracy. Adapted with permission from ref 134. Copyright 2022 Elsevier. **(D)** Scanning electron microscope images of 3D printed polymer structures reinforced with a) Fe_2_O_3_, b) ZnO, c) NiO, d) Al_2_O_3_, e) TiO_2_, f) MgO nanoparticles. Reprinted with permission from ref 135. Copyright 2022 John Wiley and Sons.

In conclusion, nanoreinforcement is highly dependent on the distribution of NPs within the polymer matrix. Well-dispersed NPs promote effective nanoreinforcement (Figure 5B-D), resulting in increased strength, while a nonhomogeneous distribution of filler NPs can lead to the formation of defects, weakening the mechanical properties.^136^ On the other hand, nanoreinforcement was also reported to mediate stress-transfer as a macroscale reinforcing mechanism for magnetic NPs directionally assembled into microstructured strings. This way, even the symmetric spherical NPs much smaller than the critical length required for stress-transfer developed effective shear stress between the matrix and filler, promoting macroscale reinforcement, despite not being covalently bonded.^137^ Additionally, photoactive MOx NPs lead to higher conversion rates in the surrounding polymer resin, which should further amplify the nanoreinforcement effect.

Several studies have investigated the (thermo)­mechanical properties of polymer nanocomposites reinforced with MOx NPs prepared using vat 3D printing technology. Selected studies and their reported enhancements and thermomechanical properties are summarized in Table 2. The improvement of mechanical properties depends on the nanofiller content; these properties decline beyond a certain critical concentration (indicated in Table 2), primarily due to insufficient curing. Furthermore, higher NP content can degrade printability to the point where vat 3D printing becomes impossible. The effect of NP loading thresholds was reported also by Mubarak et al.,^138^ who found that concentrations of silver-doped TiO_2_ (Ag-TiO_2_) NPs higher than 1.0 wt. % resulted in lower cross-linking density in polymer nanocomposites, weakening mechanical properties such as tensile and flexural strength. Somewhat higher NP loadings can be processed if homogeneous NP distribution is ensured. Additionally, using an optimized light source with higher intensity or a suitable photoinitiating system can be beneficial.^139^

**Table 2:** Summary of Enhanced Mechanical Properties and Nanoparticle Loading Thresholds in Polymer Nanocomposites with Metal Oxide Nanoparticles Prepared via Vat 3D Printing^a^ ^,^ ^a^

**Nanoparticles**	**Resin**	**Enhanced properties (values for neat resin)**	**NP critical concentration**	**ref.**
TiO_2_	Urethane-acrylate	σ_B_ = 47.4 MPa (23.4 MPa);	N/A (only 1 wt. % used)	^140^
		*H*_nano_ = 0.226 GPa (0.126 GPa);		
		*E*′ (30 °C) = 1827 MPa (1515 MPa);		
		*T*_g_ = 79.6 °C (71.7 °C)		
Ag-TiO_2_	Acrylate	σ_B_ = 44.7 MPa (27.8 MPa);	1 wt. %	^138^
		σ_f_ = 70.7 MPa (42 MPa);		
		*H*_nano_ = 0.225 GPa (0.166 GPa);		
		*E*′ (30 °C) = 1953 MPa (1495 MPa);		
		*T*_g_ = 86.5 °C (79.3 °C)		
TiO_2_	Epoxy-acrylate	σ_B_ = 47.8 MPa (25.3 MPa);	N/A (only 0.25 wt. % used)	^141^
		σ_f_ = 73.1 MPa (68.8 MPa);		
		*H* = 81.1 (76.9)		
Fe_2_O_3_, ZnO, NiO, Al_2_O_3_, TiO_2_, MgO	Epoxy	*H*_micro_ ≈ 27.6 HV0.1 with Fe_2_O_3_ (16.6 HV0.1)	N/A (only 2 wt. % used)	^135^
ZnO	Acrylate	σ_B_ = 11.7 MPa (4.8 MPa);	2.5 wt. %	^134^
		*ε*_f_ = 15% (12.8%);		
		*E*_YM_ = 408 MPa (107 MPa)		
ZnO	Acrylate	σ_B_ ≈ 54.1 MPa (48,4 MPa);	0.1 wt. %	^142^
		*E*_YM_ = 515.8 MPa (495.6 MPa)		
ZnO, Al_2_O_3_-ZnO (AZO)	Acrylate	*E*′ = 1326 MPa (892 MPa);	1 wt. % with AZO	^66^
		*T*_g_ = 60 °C (54 °C);		
		*HDT* = 41.9 °C (41.4 °C)		
Cu_2_O	Urethane-acrylate	σ_t_ = 66.3 MPa (57.8 MPa);	0.5 wt. %	^143^
		U = 4.4 kJ/m^2^ (8.6 kJ/m^2^);		
		*H*_micro_ = 24.2 HV (18.9 HV)		
Al_2_O_3_	Acrylate	*E*′ = 5985.6 MPa (1711 MPa);	1 wt. %	^144^
		U = 2.12 J/cm^3^ (1.68 J/cm^3^);		
Ag_2_O	Acrylate	σ_B_ = 60.3 MPa (69 MPa);	1 wt. %	^133^
		σ_f_ = 104 MPa (111 MPa)		
Fe_3_O_4_, CoFe_2_O_4_	Urethane-acrylate	*E*_*S*_ = 9.2 MPa (7.4 MPa);	3 wt. % with CoFe_2_O_4_	^120^
		*ε*_f_ = 39% (36.5%);		
		*T*_g_ = 20 °C (21 °C)		

a The values of mechanical properties in brackets are provided for pure resin.

b Abbreviations: Tensile strength (σ_B_), Nanohardness (*H*_nano_), Storage modulus (*E*′), Glass transition temperature (*T*_g_), Flexural strength (σ_f_), Hardness (*H*), Microhardness (*H*_micro_), Fracture strain (*ε*_f_), Young’s modulus (*E*_YM_), Heat deflection temperature (*HDT*), Tensile stress (σ_t_), Toughness (*U*), Secant modulus (*E*_S_)

### 3.2 Thermal Properties of Polymer Nanocomposites

Thermal functional properties refer to the characteristics of materials that govern their behavior in response to temperature changes, thermal energy transfer, and their ability to manage heat in various applications. These characteristics are particularly important in electronics (wearable devices, dielectric capacitors, electrical equipment),^31,145^ energy systems (thermal interface materials, battery thermal management, solar thermal storage, radiative cooling),^31,146,147^ and thermal insulation (lightweight panels, construction materials).^11,146,148,149^

Certain thermomechanical properties, such as *T*_g_ and *HDT*, were previously mentioned alongside mechanical properties due to the coupled influence of temperature and mechanical factors like stress and deformation during measurement. Despite the growing interest in vat photopolymerization-based 3D printing of metal MOx NP/polymer nanocomposites, literature addressing their thermal properties remains scarce.

Generally, polymers exhibit poor thermal conductivity due to vibrational modes being localized in amorphous regions. The heat transfer could be enhanced by promoting internal alignment or by adding thermally conductive fillers. The system is then usually dominated by the filler-matrix coupling below the percolation limit, or the interfiller contact resistance when a continuous filler network is formed (Figure 6A). Moreover, cross-linking also promotes thermal conductivity due to both bonding and nonbonding interactions, while NPs were reported to lower the heat capacity of the unreacted photopolymer mixture, supposedly due to the nanoreinforcement.^150,151^ However, literature addressing thermal properties of 3D printed metal MOx NP/photopolymer nanocomposites remains scarce, despite the growing interest in vat photopolymerization 3D printing.

**Figure 6:**
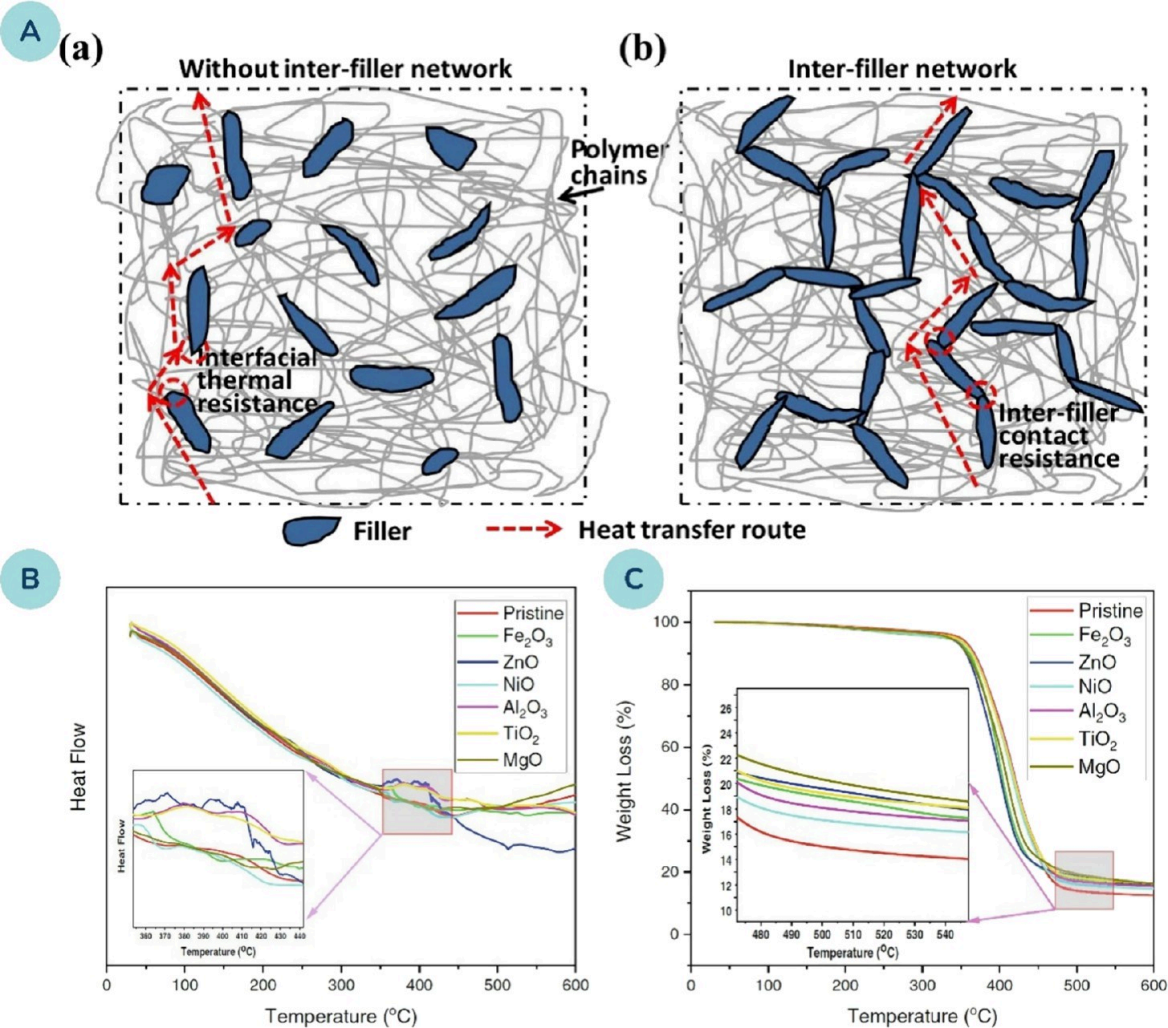
**(A)** Schematic of polymer nanocomposites without (a) and with (b) interfiller networks; thermally conductive pathways shown by dashed lines. Reprinted with permission from ref 151. Copyright 2018 Elsevier. **(B)** Differential scanning calorimetry and **(C)** thermogravimetric analysis of 3D printed polymer nanocomposites reinforced with various metal oxide nanoparticles. Reprinted with permission from ref 135. Copyright 2022 John Wiley and Sons.

The current research mostly deals with the effects of MOx NPs on the thermal stability of polymer nanocomposites. Muburak et al.^138^ investigated the thermal stability and thermal conductivity of polymer nanocomposites reinforced with Ag-TiO_2_ NPs. The results showed an improvement in thermal stability compared to the pristine polymer resin. However, the residual char value decreased at NP concentrations exceeding 1 wt. %, indicating reduced thermal stability above this limit. This reduction was attributed to potential agglomeration and poor dispersion of the NPs. A similar trend was observed for thermal conductivity, which increased steadily up to 1 wt. % of Ag-TiO_2_ NPs but slightly declined at higher concentrations. The maximum measured thermal conductivity showed a 40.2% increase compared to the unfilled resin.

Thermal stability was also investigated by Aktitiz et al.,^135^ who examined various MOx NPs at a concentration of 2 wt. %, as shown in Figure 6B, C. Differential scanning calorimetry (DSC) revealed that the polymerization reaction in the nanocomposite samples was not fully completed due to two primary factors (Figure 6B). First, the incorporation of MOx NPs created a two-phase heterogeneous system with differing refractive indices, causing light scattering during curing. Second, diffusion was restricted by early vitrification. These effects were further amplified by significant interfacial thermal resistance between the NPs and the polymer matrix, as well as variations in thermal conductivity levels among the different metal oxides.

Thermogravimetric analysis (TGA) showed that thermal decomposition began at approximately 350 °C and was completed by 475 °C (Figure 6C). The presence of MOx NPs in the polymer matrix enhanced thermal stability, and the residual char value increased with the addition of NPs. However, the maximum degradation temperature remained largely unchanged, primarily due to poor dispersion and aggregation of the MOx structures, which weakened the interactions between the polymer matrix and the NPs.

Garcia et al.^142^ reported similar findings when investigating ZnO NPs. Their results demonstrated that ZnO NPs delayed the onset of thermal decomposition and increased the residual char content. Notably, the influence of ZnO concentration on thermal improvements was more pronounced than the effect of increased curing temperature.

Petousis et al.^143^ also observed a significant impact on thermal performance in nanocomposites reinforced with Cu_2_O, which affected the thermal stability of 3D-printed materials above 0.5 wt. %. Higher Cu_2_O content (1 wt. % and 2 wt. %) was associated with incomplete or low-quality photopolymerization, likely due to the filler interfering with the curing process. On the other hand, Raj et al.^144^ investigated the effect of Al_2_O_3_, while the results indicated that the maximum decomposition temperature remained largely unaffected by increasing Al_2_O_3_ content. In the contrary, nanofillers rich in hydroxyl (−OH) and carboxyl (−COOH) surface functional groups may even compromise the thermal stability of polycondensation polymers due to catalytic hydrolysis.^152^

### 3.3 Electrical and Dielectric Properties of Polymer Nanocomposites

The electrical and dielectric performance of polymer nanocomposites is highly dependent on the presence of nanofiller particles. Specifically, metal oxide/polymer nanocomposites with fillers smaller than 100 nm that are homogeneously dispersed can significantly enhance electrical properties, such as dielectric strength, electrical conductivity, and voltage endurance. Such materials are developed for use in various applications, including power electronics (capacitors and energy storage applications),^153–155^ high-voltage insulation,^136,156^ and flexible electronics (wearable devices)^153,157,158^ due to their enhanced dielectric properties and mechanical flexibility.

According to percolation theory, electrical conductivity and dielectric constant are significantly improved as the quantity of conductive NPs approaches the critical concentration, known as the percolation threshold. During the percolation, there is considerable change in certain physical properties within a quite narrow concentration range. In composites comprising of insulating polymer matrix and conductive particles, the change in conductivity is explained by the formation of a conductive network within the sample, occurring when sufficient contacts are established between the conductive particles in the percolation zone.^136,159,160^ However, it is necessary to point out that the percolation depends on the dispersion of nanofillers, and not all physical properties exhibit the same percolation threshold. Sevriugina et al.^76^ reported a disparity between the electrical and rheological percolation of high-permittivity resins, while similar trends for both properties were observed in low-permittivity materials.

Several studies have examined the electrical properties of MOx NPs in relation to the percolation threshold. Rahman et al.^161^ investigated Fe_2_O_3_, TiO_2_, and NiFe_2_O_4_ NPs at 5 wt.% in a polyester resin and found that all NPs increased electrical conductivity, indicating that the percolation threshold was either reached or exceeded. Sampreeth et al.^162^ synthesized polyaniline/cerium-doped TiO_2_ (Ce-TiO_2_) nanocomposites and observed maximum electrical conductivity at 7 wt. % NP loading, attributing the percolation behavior to strong interfacial interactions that enhanced NP orientation and thereby improved the quality of the electrical network formation. Hong et al.^163^ reported percolation thresholds in low-density polyethylene nanocomposites with ZnO NPs at 14 vol. %, while micron-scale ZnO required 30 vol. % to reach the same threshold. This difference was linked to the reduced interparticle distance in the nanocomposites, which facilitated conduction via tunnelling.

Unlike the previously mentioned studies that did not employ photopolymerization, Uřičář et al.^164^ utilized SLA 3D printing to prepare polymer nanocomposites containing Fe_3_O_4_ nanoparticles at concentrations of 0.5, 1, 3, and 5 wt. %. The measured electrical resistivity was lower at all concentrations compared to pure resin, and resistivity further decreased with increasing NP concentration. However, the authors suggest that the percolation threshold had not yet been reached and likely requires an even higher nanofiller content.

Also, the influence of ZnO and AZO (alumina-doped ZnO) NPs on the electrical behavior of photopolymer nanocomposites has been studied.^66^ The percolation threshold for both NP types was identified at approximately 1 vol. %, with minimal contribution to conductivity below this concentration. A noticeable decrease in resistivity occurred at 2 vol. %, although the measured resistivities remained relatively high. The nanocomposites still fell within the range of insulating materials, with only the samples containing 2 vol. % of ZnO and AZO nanoparticles reaching the borderline between antistatic and insulating classifications.

Magnetic NPs Fe_3_O_4_ and CoFe_2_O_4_ also have a significant impact on electrical properties.^120^ The incorporation of 6.3 wt.% Fe_3_O_4_ led to a two-order-of-magnitude increase in electrical conductivity compared to the pristine resin, while 3 wt.% CoFe_2_O_4_ produced a similar effect. However, iron oxide-based magnetic particles should not influence the electrical conductivity of magnetic nanocomposites. Thus, the increase in electrical conductivity is attributed to charges at the particle–polymer interfaces and ionic conductivity contributions of the polymer matrix, which vary with the filler content and degree of curing.

Overall, there is a lack of research on vat 3D printing of polymer nanocomposites with MOx NPs specifically for improving conductivity. This is because MOx NPs generally have much lower intrinsic conductivity compared to other NPs such as carbon or metals. These other NPs reach their percolation threshold at relatively low concentrations, making them more suitable for electrical applications.^11^ However, the dielectric properties of MOx NP/polymer nanocomposites show significant potential and warrant further exploration. Metal oxides are classified as high-dielectric-constant (high-*k*) materials, which makes them highly suitable for charge storage applications such as high-density memory, capacitors, and supercapacitors.^165^

The impact of metal oxides on the dielectric properties of polymer matrices has been extensively studied. However, only a limited number of studies have investigated the use of vat 3D printing for the fabrication of high-*k* nanocomposites.

Dielectric properties are typically assessed through dielectric permittivity, which correlates directly with the material’s capacitance, reflecting its charge-storage capacity and dielectric performance in 3D-printed nanocomposite resins.^166^ The addition of ZnO nanoparticles generally increased the complex permittivity.^66^ However, some samples, especially after postcuring, showed complex permittivity comparable to or even lower than the unfilled matrix. This behavior is attributed to the restriction of polarization caused by the adsorption of polar species on the nanoparticle surface^64^ and the nanoconfinement effect, which reduces polymer chain mobility near NPs.^62,167^

Another research by Malas et al.^168^ focused on preparing a high dielectric permittivity ceramic/polymer composite feedstock for vat 3D printing using nanosized TiO_2_ and microsized calcium copper titanate (CCT; CaCu_3_Ti_4_O_12_), as shown in Figure 7. The inclusion of both types of NPs increased permittivity compared to the base resin (Figure 7B), with TiO_2_ NPs being more effective in enhancing the relative dielectric permittivity.

**Figure 7:**
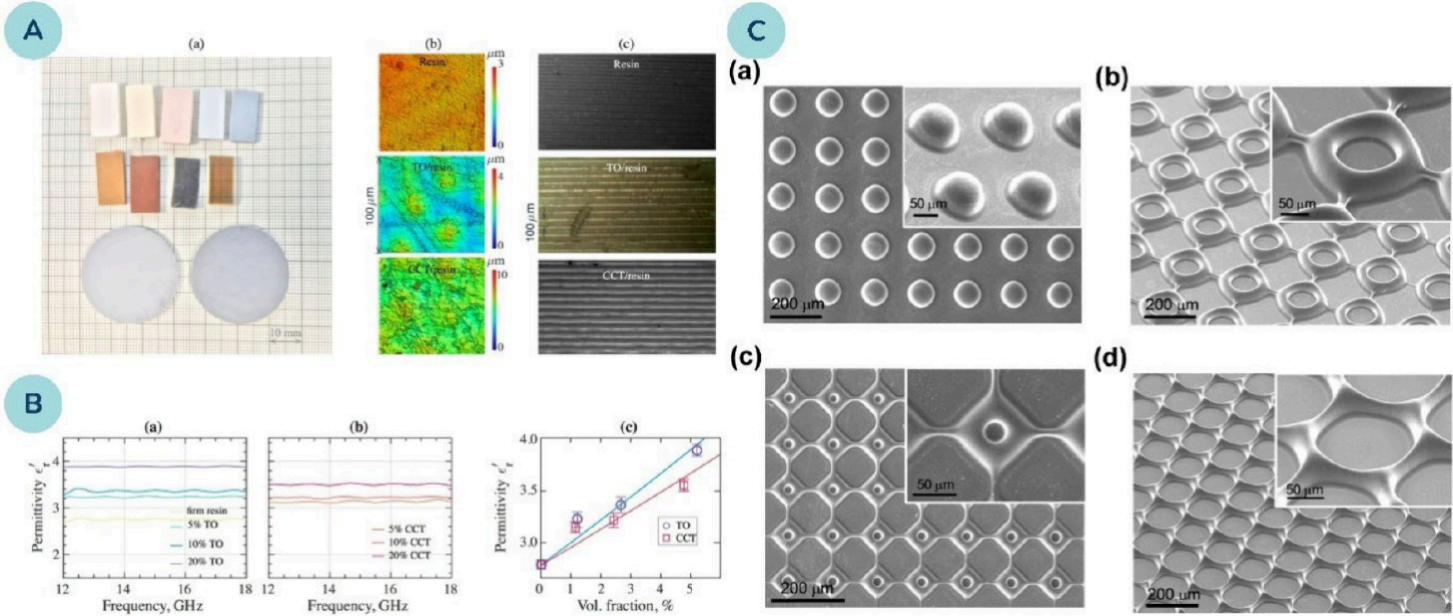
**(A)** 3D-printed samples with varying TiO_2_ nanopowder (TO) and calcium copper titanate (CCT) loadings: (a) appearance as a function of filler and resin type, (b) xy-plane surface topography of unfilled and 10 wt. % composites, and (c) zx-plane cross-sections showing build layers. **(B)** Relative permittivity of printed samples with (a) TO and (b) CCT fillers, and (c) average permittivity versus filler volume fraction (concentrations in (a, b) given in wt. %). Reprinted from ref 168. Available under a CC-BY 4.0 license. Copyright 2019 Malas et al. **(C)** Collage of microstructures (dot, square, and honeycomb arrays) fabricated by digital projection printing. Reprinted with permission from ref 169. Copyright 2014 American Chemical Society.

Additionally, certain metal oxides not only influence electrical and dielectric properties but also exhibit piezoelectric characteristics – the ability to convert compressive or tensile stress into electric charges. Prominent examples include lead zirconate titanate and barium titanate, which are essential components in devices such as acoustic imaging systems, energy harvesters, and actuators. Kim et al.^169^ used barium titanate (BaTiO_3_) to prepare piezoelectric materials through digital projection printing (Figure 7C). They chemically modified the NPs with acrylate surface groups to form direct covalent linkages with the cured resin. The study found that chemically modified BaTiO_3_ NPs at a 10 wt. % loading exhibited piezoelectric coefficients over 10 times larger than those of unmodified samples. This research not only demonstrates the feasibility of 3D piezoelectric fabrication but also highlights the creation of highly efficient piezoelectric materials through nanointerfacial tuning.

### 3.4 Magnetic Properties of Polymer Nanocomposites

Recently, vat 3D printing has been increasingly applied in magnetic property-related applications, including microactuators, fluidics, strain sensors, and functionally graded materials, as illustrated by representative examples in Figure 8.^74,170^ These smart polymer nanocomposites are responsive to external stimuli. Typically, magnetite (Fe_3_O_4_) NPs are used to impart magnetic properties to these composites. Magnetite NPs smaller than 20–30 nm were found to achieve the best performance due to their superparamagnetic nature, making it crucial to ensure a homogeneous distribution without agglomerates or aggregates. Magnetic nanocomposites have a broad range of applications in catalysis, where magnetic fluid systems facilitate processes such as wastewater treatment and pollutant remova;^74,171^ in biomedical technologies, where they are explored for magnetic resonance imaging (MRI), tissue engineering, and targeted therapies;^172–175^ and in data technologies, where their tunable magnetic response enables use in data storage devices and magneto-optical applications.^173,176^

**Figure 8:**
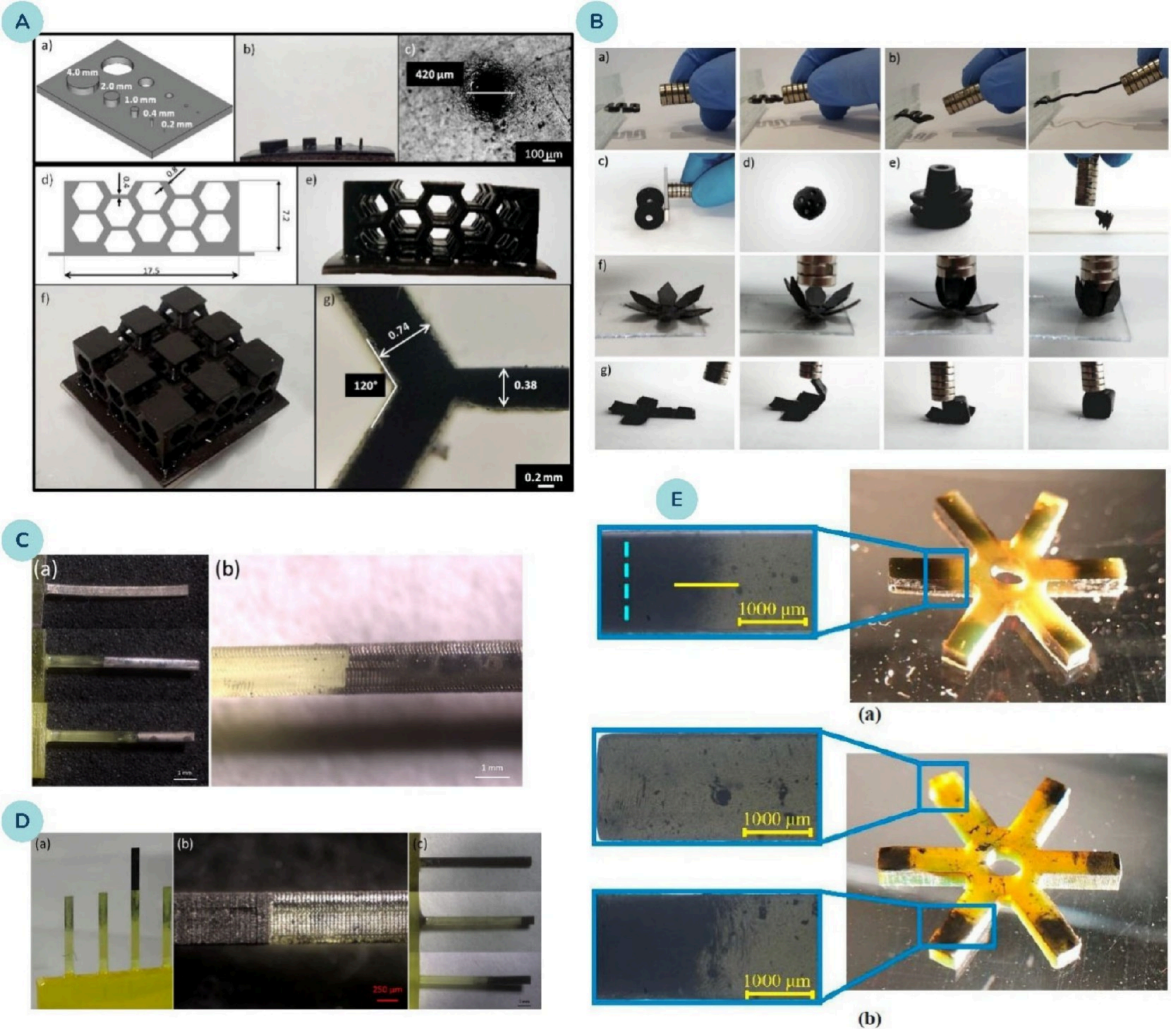
**(A)** XY-plane resolution of photocurable resins loaded with magnetite nanoparticles: (a–c) CAD design, printed object, and optical micrograph of holes and pillars of decreasing size (scale bar 100 μm). (d–f) CAD model and corresponding 3D-printed honeycomb structure showing high fidelity to design. (g) Optical detail of hexagons (scale bar 200 μm). **(B)** 3D-printed magnetic objects: (a) spring with 2 wt% magnetite; (b) spring with 6 wt. % magnetite; (c) wheels; (d) sphere; (e) cone-like structure; (f) flower closing under a magnetic field; (g) 2D flexible–rigid structure forming a 3D cube upon magnetic actuation. Reprinted with permission from ref 177. Copyright 2019 John Wiley and Sons. **(C)** (a) Cantilever-based magnetic structures from photopolymers loaded with magnetite nanoparticles, fabricated by SLA printing with selective tip plating. (b) Metallization length controlled by immersion depth, with capillary effects creating a wavy plated–bare interface. **(D)** (a) Array of cantilevers prepared from two photopolymerizable resins containing magnetite nanoparticles. (b) High-resolution features with micrometric control at resin interfaces. (c) Samples with varied magnetic part lengths. Reprinted with permission from ref 178. Copyright 2016 American Chemical Society. **(E)** Examples of 3D printed parts with graded distributions of magnetite nanoparticles: (a) using a tubular magnet, (b) using a circular array. Reprinted with permission from ref 179. Copyright 2019 Elsevier.

So far, a few research articles have been published on the use of vat 3D printing to produce magnetic nanocomposites. Among these, Lantean et al.^177^ prepared magnetoresponsive polymer nanocomposites using DLP with Fe_3_O_4_ NP content up to 6 wt. % (Figure 8A, B). They found that the magnetization of the samples was not affected by the formulation of the polymer resin and was simply proportional to the NP concentration. While high-resolution 3D prints were achievable at 6 wt. % NP concentration, increasing the concentration to 8 wt. % resulted in reduced light absorption by the PI due to the presence of NPs, leading to lower curing rates and compromised mechanical properties.

Similarly, Credi et al.^178^ utilized SLA 3D printing to prepare cantilever-based magnetic microstructures, incorporating Fe_3_O_4_ NPs in concentrations ranging from 1 to 4 vol. % (Figure 8C, D). The magnetic sensitivity of the nanocomposite structures was characterized by tip deflection behavior when subjected to an external magnetic field. The results showed that samples entirely printed with the ferromagnetic NPs exhibited larger deformations compared to those with a photopolymer selectively coated with a metal layer via an electroless plating process. Thus, the incorporation of Fe_3_O_4_ NPs has proven to be an effective method for creating novel smart magnetic photopolymers.

Löwa et al.^180^ utilized two types of magnetic NPs: an aqueous suspension of carboxydextran-coated iron oxide NPs, which included both single-core and multicore magnetic NPs, and a light hydrocarbon oil-based ferrofluid. The study evaluated the feasibility of printing magnetic nanocomposites, focusing on the homogenization process, mixture ratios, long-term stability, and processability of the final composites. The fabricated nanocomposites were successfully used as long-term stable phantoms for magnetic particle imaging (MPI) applications.

Another approach was introduced by Lu et al.^181^ who developed the magnetic field-assisted projection stereolithography (M-PSL). In this method, a controlled amount of nano- or microsized ferromagnetic particles is deposited into a liquid photopolymer using a programmable microdeposition nozzle. An external magnetic field is then applied to guide the magnetic particles into predefined positions, aligning them into specific orientations and patterns. Subsequently, a digital mask image is employed to selectively cure the photopolymer, ensuring that the magnetic particles are fixed in their desired distribution. This technique enables precise control over the magnetic particle filling rate, spatial distribution, and structural arrangement, facilitating the fabrication of smart materials with complex and heterogeneous functionalities.

As a next step, Safaee et al.^179,182^ invented a novel DLP-based method for fabricating functionally graded materials by applying a magnetic field during the 3D printing of polymer nanocomposites with Fe_3_O_4_ NPs (Figure 8D). Different distributions and gradients of the magnetic particles were achieved by varying the layout of the magnets or the distance from the magnet to the print bed. As a result, it was found that a stronger magnet can locally increase particle concentration and create a higher gradient of particle distribution in the transition area. This method is gaining widespread recognition and is being increasingly utilized in recent studies.

In contrast to the previously mentioned studies using magnetite, Aktitiz et al.^183^ used α-Fe_2_O_3_ NPs, which have a lower magnetic susceptibility. By measuring the hysteresis curves, it was found that the polymer matrix itself is strongly diamagnetic, and low NP content (up to 0.5 wt. %) did not significantly alter the magnetic properties. However, a change was observed at 1 wt. % of α-Fe_2_O_3_ NPs, where the maximum magnetization magnitude increased by more than four times compared to the pure polymer matrix.

Although most studies use Fe_2_O_3_, particularly in the form of magnetite (Fe_3_O_4_), to achieve magnetic properties, some also utilize other nanofillers. Mendes-Felipe et al.^120^ studied not only Fe_3_O_4_ but also CoFe_2_O_4_ (CFO). Magnetic properties were evaluated by measuring the hysteresis loop using a vibrating sample magnetometer (Figure 9). The results demonstrate that the magnetic particles did not undergo any degradation or oxidation when incorporated into the polymer matrix. By varying the particle type and content, different values of saturation magnetization, coercivity, and remanence were obtained. Regarding the evolution of magnetic properties with increasing filler concentration, a proportional trend was observed in all samples. Thus, nanocomposites with Fe_3_O_4_ exhibited a saturation magnetization of up to 3.70 emu/g, a remanence of 0.27 emu/g, and zero coercivity. In contrast, nanocomposites containing CFO, showed an increased saturation magnetization of up to 6.50 emu/g, a remanence of 1.69 emu/g, and a coercivity of 2000 Oe.

**Figure 9:**
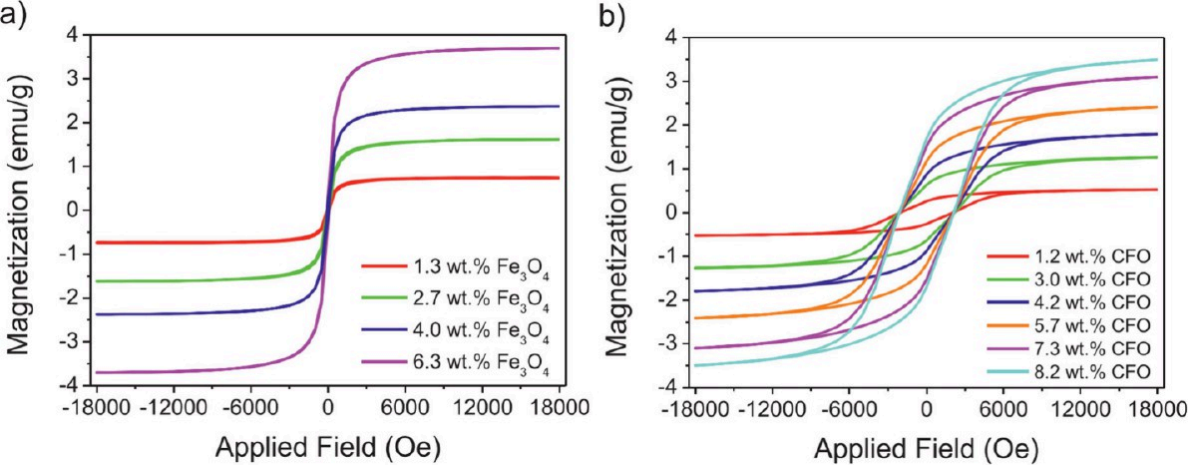
Room-temperature magnetic hysteresis loops of (a) Fe_3_O_4_/polymer nanocomposites and (b) CoFe_2_O_4_ (CFO)/polymer nanocomposites, measured using a vibrating sample magnetometer. Reprinted with permission from ref 120. Copyright 2021 Elsevier.

### 3.5 Optical Properties of Polymer Nanocomposites

The key optical properties of polymer nanocomposites include light absorption, refraction, transmission, reflection, and scattering, which are primarily influenced by the material composition, atomic and molecular arrangement, and the shape and size of nanofillers. In particular, MOx NPs play a critical role in modifying these properties, as their nanoscale size and high refractive indices significantly impact light interaction. Due to the quantum confinement effect, where the particle size is smaller than the wavelength of interacting light, nanocomposites exhibit unique optical behaviors that differ from their bulk counterparts.^136,184^ The application areas of these materials extend from optoelectronic devices, such as photodetectors and phototransistors,^185–187^ to thin films for chemical sensing, energy storage, and power management.^188,189^

Several studies have demonstrated the feasibility of vat photopolymerization 3D printing for fabricating polymer-based optical nanocomposites with MOx NPs, offering tailored optical functionalities. For example, Bisht et al.^190^ utilized 3D printing to create polyurethane-acrylate resin with ZnO NPs for wide spectral photoresponse optical detectors (Figure 10A, B). The focus was on preparing UV photodetectors, which have applications in various fields such as deep space communication, flame detection, solar flare detection, ozone sensing, and biochemical detection. Wide band gap semiconductors like ZnO are prominent materials for UV detection. This research confirmed the feasibility of vat 3D printing for ZnO/polymer composite photodetectors, with the prepared device demonstrating a better photoresponse time compared to similar devices with ZnO.

**Figure 10:**
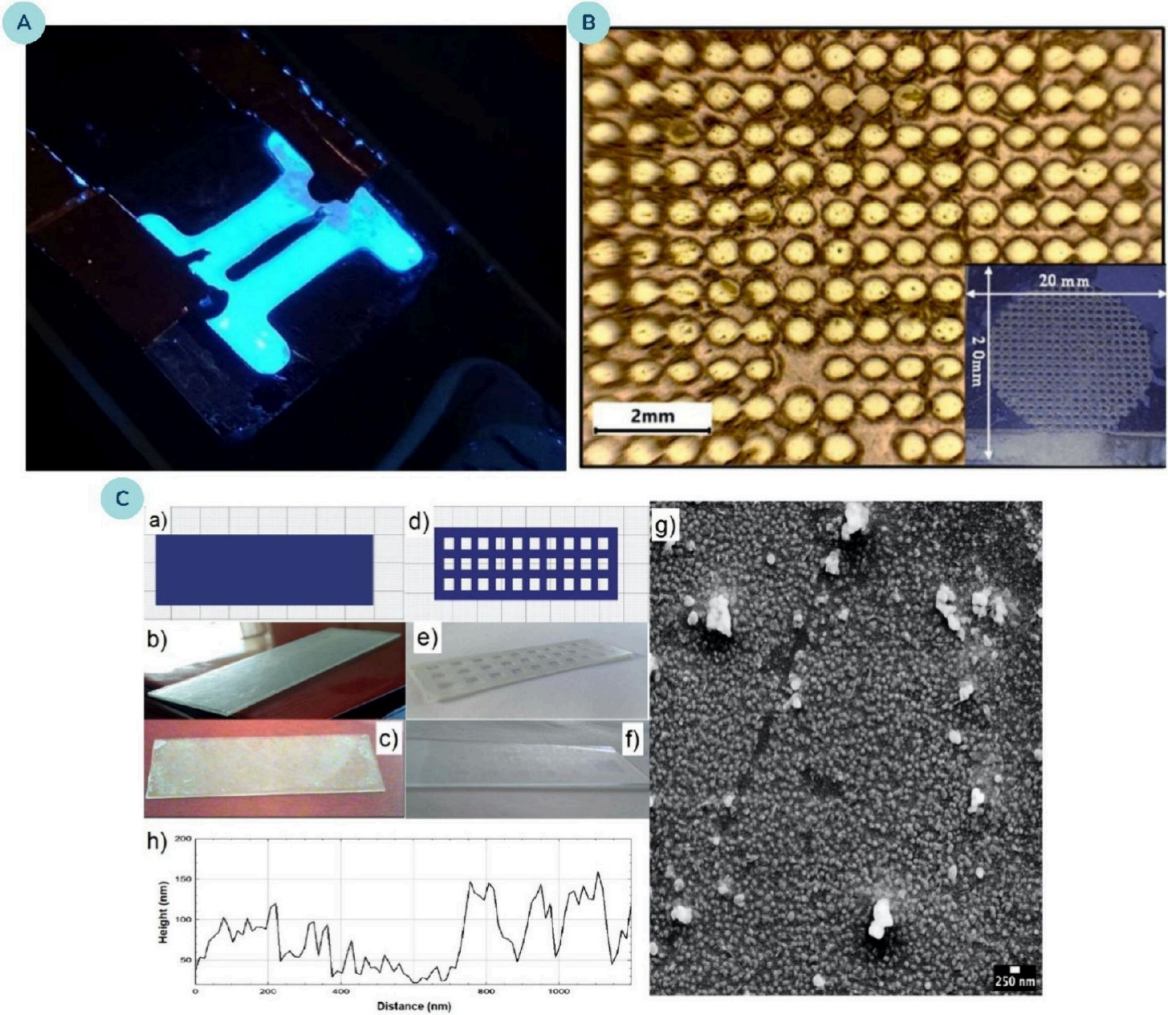
**(A)** Photodetector fabricated by DLP 3D printing of a resin/ZnO composite (10 wt. % ZnO) on a silicon substrate under 370 nm illumination. **(B)** Optical image of an array of resin/ZnO composite dots produced by DLP 3D printing. Adapted with permission from ref 190. Copyright 2023 Elsevier. **(C)** TiO_2_ nanoparticle thin films fabricated by DLP: (a) rectangular CAD design, (b) printed resin/TiO_2_ film, (c) calcinated film, (d) mesh CAD design, (e) printed resin/TiO_2_ mesh, (f) calcinated mesh showing air bubbles from resin boiling during calcination, (g) SEM micrograph, (h) profile analysis. Reprinted with permission from ref 191. Copyright 2021 Elsevier.

Zhang et al.^192^ developed a unique NiO-doped silica glass using DLP 3D printing technology. They introduced nickel­(II) acetylacetonate as a precursor during the 3D printing process, which thermally decomposes to form NiO NPs. Subsequent heat treatment results in a homogeneous distribution of NiO NPs within the silica glass, exhibiting optical nonlinear effects that can be harnessed in a range of optoelectronic devices.

In a study aimed at broadening the optical capabilities of polymer systems, Weber et al.^193^ developed a special nanoink composed of a commercial photopolymer and zirconium dioxide (ZrO_2_) NPs. The goal of this study was to expand the limited range of optical properties achievable with conventional polymer systems. By enhancing the attainable optical properties, polymer-based optical systems can be used in various advanced applications, such as smartphone cameras. The study found that the refractive index of the nanoink could be controlled by varying the ZrO_2_ NP content (up to 20 vol. % in this study). Subsequently, two-photon direct laser writing was used to 3D print simple optical devices (an optical lens and a multimaterial achromatic doublet). These devices demonstrate that nanocomposites can serve as next-generation optical materials, offering unprecedented design freedom.

Another promising category utilizing the optical properties of polymer nanocomposites is thin films, which are thin layers of solid material ranging in thickness from a few nanometers to hundreds of microns. Thin films are employed across various technological fields, including supported catalysts, solar cells, transistors, diodes, sensors, and thermoelectric devices. Vat 3D printing can also be used to prepare these thin films (Figure 10C), as first described by Gonzalez-Ocaguenda et al.^191^ In this study, TiO_2_ was used as a nanofiller in a UV photoresin at a ratio of 1:10. Optical analysis revealed that the thickness of the prepared films varied between 80 and 120 nm, with the films consisting of a single layer of TiO_2_ NPs on the substrate.

In conclusion, the advancements in polymer nanocomposites not only enhance the functionality of optical devices but also open up new possibilities for integrating these materials into next-generation technological applications.

### 3.6 Biofunctional Properties of Polymer Nanocomposites

Polymer nanocomposites incorporating MOx NPs are crucial in various biomedical applications, including diagnostics, drug delivery, medical implants, magnetic resonance imaging (MRI), tissue engineering, and cancer treatment.^130,165,194,195^ There is also a growing demand for the preparation of biofunctional materials through vat 3D printing, as it serves as a powerful tool for advancing biological applications, particularly in creating custom, high-resolution, biocompatible structures suitable for personalized medicine.^196,197^ Given their broad utility, MOx NPs/polymer nanocomposites have been extensively explored for their biofunctional applications in biomedical fields.

This review highlights key applications where vat 3D printing enhances the biofunctionality of these nanocomposites. However, the widespread adoption of vat 3D printing in biomedical applications is hindered by the cytotoxicity of photosensitive resins and photoinitiators commonly used in the process.^198^ To overcome these limitations, researchers are developing alternative biocompatible materials with improved safety profiles.^197^ Furthermore, advancements in water-soluble photoinitiator NPs have contributed to reducing cytotoxicity, expanding the potential of vat 3D printing for biomedical applications.^199,200^

Among the many biomedical applications, dentistry has been a major focus for leveraging the biofunctional properties of MOx NPs. In dental materials, MOx NPs serve multiple functions beyond structural reinforcement; they release ions to combat oral pathogens, deliver calcium phosphate compounds, enhance imaging contrast, protect dental tissues from bacterial acid attacks, and improve the mineral content of the bonding interface.^201^ Studies have shown that TiO_2_, ZrO_2_ and Al_2_O_3_ improve dental material durability by preventing denture fractures and reducing degradation from oral fluids. These benefits are primarily due to their ability to increase impact strength and fracture toughness while significantly reducing water sorption.^202,203^ Among these metal oxides, ZnO NPs stand out in dentistry due to their antimicrobial, regenerative, and mechanical properties. Their antimicrobial action involves NP degradation in the acidic lysosomal environment, converting core metals into ions and releasing toxic substances that disrupt microbial reproduction. Other antimicrobial mechanisms include local environmental changes near microbes and the generation of reactive oxygen species (ROS), as shown in Figure 11.^204,205^ Since photopolymerization is a fundamental process in dental practice, numerous reviews have extensively examined this topic.^206–209^

**Figure 11:**
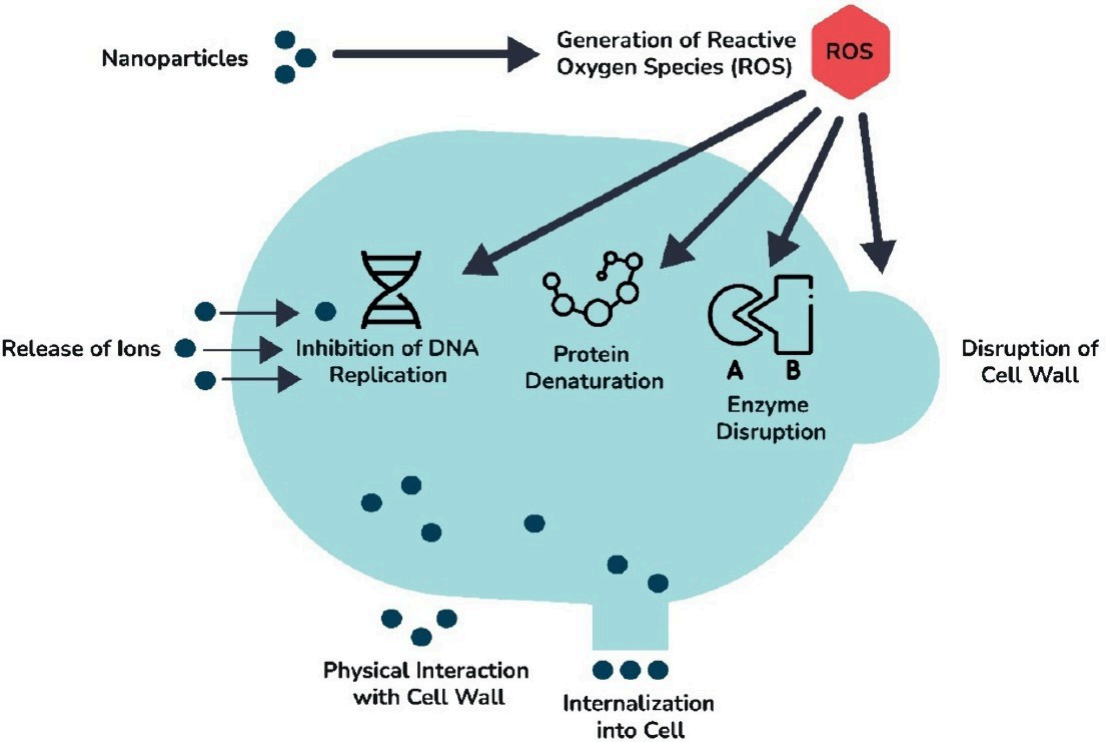
Visualization of antimicrobial mechanisms of metal oxide NPs. Adapted from ref 205. Available under a CC-BY 4.0 license. Copyright 2017 Wahid et al.

Beyond dental applications, vat 3D printing has also been explored for antimicrobial materials. Petoutis et al.^143^ developed photopolymerizable resins incorporating Cu_2_O NPs for SLA 3D printing, specifically designed to inhibit or eliminate bacteria (Figure 12A, B). Cu_2_O NPs were incorporated at concentrations of 0.5, 1, and 2 wt. %. At concentrations above 0.5 wt. %, Cu_2_O NPs tended to agglomerate, adversely impacting polymerization quality. Despite this, higher NP concentrations enhanced antibacterial properties.

**Figure 12:**
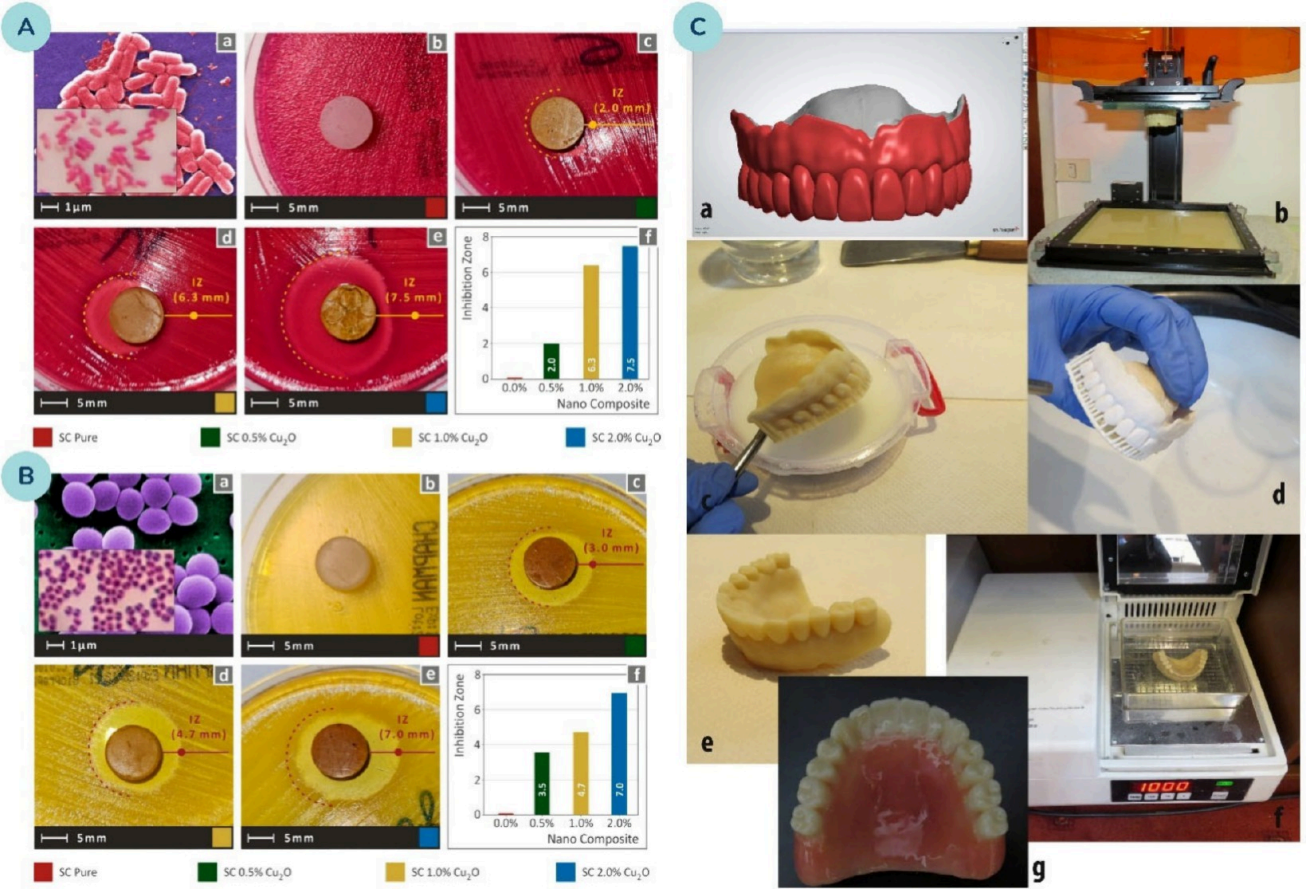
Biocidal performance of Cuprous Oxide–filled nanocomposites: Panels **(A)** *E. coli* and **(B)** *S. aureus* show experiments performed with the respective bacteria: (a) reference bacterial morphology; (b–e) Petri dish images after 24 h cultivation with nanocomposites containing increasing Cu_2_O loadings, illustrating progressive inhibition zones; (f) comparative plots of inhibition zone diameter as a function of Cu_2_O concentration (wt. %). Reprinted from ref 143. Available under a CC-BY 4.0 license. Copyright 2022 Petoutis et al. **(C)** Design and fabrication workflow for stereolithographic manufacturing of complete dentures incorporating TiO_2_ nanoparticles: (a) virtual denture design; (b) printed construction attached to the build platform; (c) cleaning of the denture in isopropanol; (d) drying and removal of printing supports; (e) polished prototype denture; (f) final postcuring in a light-curing; (g) esthetic adjustment of the final product. Reprinted with permission from ref 210. Copyright 2017 Elsevier.

Similarly, MOx NPs have been incorporated into hydrogels, which are widely studied in tissue engineering for their ability to replicate the extracellular matrix of natural tissues when implanted.^197^ Mpuhwe et al.^211^ prepared CeO_2_-incorporated hydrogel using DLP 3D printing. The resulting 3D-printed hydrogels demonstrated excellent antimicrobial efficacy due to the presence of CeO_2_ NPs, while also exhibiting favorable mechanical properties, a high swelling ratio, and a desired biodegradation rate.

Turning to another MOx NPs, Totu et al.^210^ incorporated TiO_2_ NPs into a commercial poly­(methylmethacrylate) (PMMA) stereolithographic resin to manufacture 3D-printed dental prostheses (Figure 12C). The addition of TiO_2_ NPs to the PMMA matrix demonstrated antibacterial effects, particularly against *Candida* species. An optimal NP concentration of 0.4 wt. % TiO_2_ was identified, providing both good antibacterial properties and high printing quality.

In a related study, Chen et al.^212^ developed a TiO_2_/polymer nanocomposite with enhanced antibacterial and biocompatibility characteristics specifically for vat 3D printing of dental prostheses. In this study, the TiO_2_ NPs were functionalized with carboxylic acid to improve their morphology and dispersion within the polymer resin. As a result, the functionalized TiO_2_ NPs were uniformly dispersed without agglomeration, achieving a 99% killing efficiency against bacterial pathogens *Escherichia coli* and *Staphylococcus aureus* under dark conditions.

Shannon et al.^133^ investigated the suitability of Ag_2_O as an antimicrobial filler for vat 3D printing. Even at low concentrations of 1 wt.%, Ag_2_O exhibited strong antimicrobial activity against *S. epidermidis*. However, lower concentrations behaved similarly to the unfilled resin, allowing bacteria to survive direct contact, suggesting a minimum threshold of Ag_2_O required in 3D-printed samples to effectively inhibit bacterial growth.

Beyond their antimicrobial properties, MOx NP/polymer nanocomposites also play a crucial role in drug, gene, stem cell, and imaging agent delivery systems, enabling precise release and controlled biodegradation within physiological environments. A well-established approach in this field is the fabrication of magnetic micromachines via single- and two-photon polymerization (TPP), as shown in Figure 13. Numerous studies^213–216^ have developed magnetic polymer nanocomposites incorporating Fe_3_O_4_ nanoparticles. These systems are designed to absorb and release biologically active substances and degrade in aqueous physiological environments, facilitating noninvasive excretion of degradation products and providing an alternative to manual or surgical retrieval. Expanding these developments, Ceylan et al.^217^ employed a micro–TPP process to fabricate personalized magnetic micromachines using patient-derived blood biomaterials, aiming to mitigate biocompatibility concerns. They created a precursor mixture comprising blood components - plasma, serum albumin, and platelet lysate - along with rose bengal as a photosensitizer and Fe_3_O_4_ NPs serving as magnetic transducers for externally powered propulsion. These micromachines respond to external magnetic fields for controlled movement and exhibit pH-responsive shape memory behavior, facilitating precise drug delivery and release.

**Figure 13:**
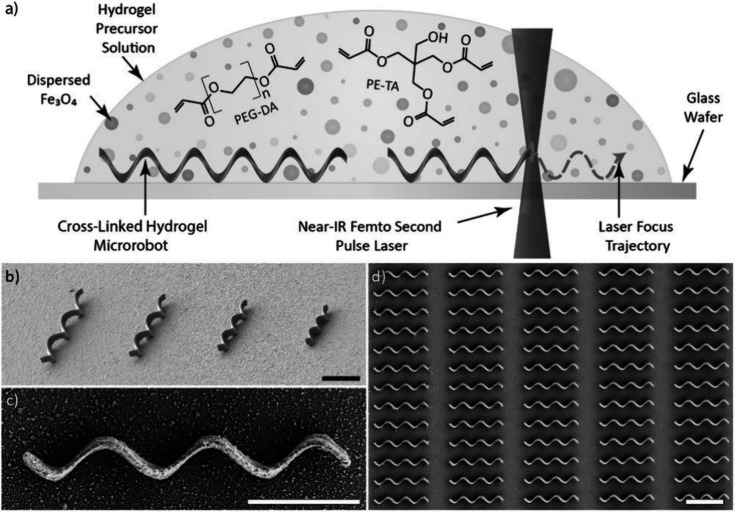
**(a)** Two-photon polymerization fabrication of superparamagnetic hydrogel microrobots from Fe_3_O_4_-loaded nanocomposite; **(b)** tilted SEM image of helical test patterns (1.5–3.0 μm diameter, 14–28 μm length); **(c)** SEM top view of a single helical microrobot; **(d)** SEM top view of an array of microrobots. Scale bars: 10 μm. Reprinted with permission from ref 213. Copyright 2015 John Wiley and Sons.

TPP is also utilized in fabricating various medical devices such as microneedle arrays and cell culture devices due to their biocompatibility, ultralow shrinkage, and allowance for cell and biomolecule adhesion.^218^ For instance, Marino et al.^219^ fabricated structures suitable for cell culturing, which can serve in bone tissue engineering, by incorporating piezoelectric BaTiO_3_ NPs into a commercial resin. Conversely, Skoog et al.^220^ utilized ZrO_2_ NPs to fabricate scaffolds designed for long-term stem cell cultivation.

## 4 Conclusions

Vat photopolymerization has emerged as a powerful technique for fabricating polymer nanocomposites with tailored functional properties, enabled by the incorporation of semiconducting metal oxide NPs. These NPs not only enhance mechanical, thermal, electrical, magnetic, optical, and biofunctional performance but also influence the photopolymerization process through their photocatalytic activity and interaction with curing light. This dual role creates unique opportunities for multifunctional materials while also introducing additional complexity into nanocomposite design and processing.

Overall, a critical evaluation of the current state of the field reveals that, despite the clear multifunctional benefits of incorporating MOx NPs into vat photopolymerization, their practical use is still constrained by fundamental processing and performance challenges. Addressing these limitations requires targeted strategies, including advanced dispersion control such as chemical surface modification or dispersing agents, resin formulation with optimized photoinitiator systems to balance light absorption, and the possible implementation of hybrid nanofiller systems, where MOx NPs are integrated with carbon-based or ceramic nanomaterials to broaden functionality.

In the near term, research should prioritize strengthening fundamental printability and functional performance by improving dispersion stability and refining system composition. These developments will establish the foundation required for future application-driven advances, ultimately enabling vat photopolymerization to compete with conventional processing routes in producing functional MOx NP/polymer nanocomposites.

In this context, the present review provides a comprehensive basis for the rational design and development of MOx NPs/polymer nanocomposites fabricated via vat photopolymerization, supporting their future implementation in a wide range of high-performance and biomedical applications. Importantly, advancing vat photopolymerization for MOx NP/polymer nanocomposites could also expand its adoption into fields where such materials are already utilized but are currently manufactured through alternative processing routes. By enabling precise control over dispersion, curing, and functional performance, vat photopolymerization has the potential to open new pathways for integrating these nanocomposites into applications that have so far remained inaccessible to this additive manufacturing technology.
